# Percentage Ratios of Cutting Forces during High-Reed Face Milling

**DOI:** 10.3390/ma16010384

**Published:** 2022-12-31

**Authors:** Martin Reznicek, Cyril Horava, Martin Ovsik

**Affiliations:** Faculty of Technology, Tomas Bata University in Zlin, Vavreckova 5669, 760 01 Zlín, Czech Republic

**Keywords:** milling, cutting force, high-feed milling, depth of cut

## Abstract

This research paper is concerned with the experimental study of high-feed end milling of 1.4541 (X6CrNiTi18-10) stainless steel with replaceable cermet plates. Several machining operations were performed under different cutting conditions. The variable values were depth of cut, feed per tooth and cutting speed. The results were analyzed, and cutting forces were evaluated for dependence on cutting conditions (cutting speed, depth of cut, feed per tooth). The obtained data were statistically processed and plotted in graphs. It was found that the percentage distribution of cutting forces changed as the tool load increased. The ratio of forces acting in individual axes also changed with varying trends. An increasing trend was recorded in the x and y axes, while a decreasing trend was recorded in the z axis. Measured change, approximately 10%, can no longer be neglected as it can significantly influence the clamping stability of a part.

## 1. Introduction

Manufacturing processes, especially machining, can influence the surface integrity of a workpiece due to the high temperatures produced during cutting, which result in plastic deformation in the workpiece (residual stresses), changes in surface geometry (roughness, cracks, distortion) and chemical reactions between the tool and workpiece [1].

Many articles deal with the prediction and study of cutting forces. Wen and his team [2] invented a cutting force prediction program. Grossi et al. [3] dealt with the shapes of cutting forces. Wang [4] identified and analyzed the cutting force coefficients in the helical milling process. Popovic [5] performed experimental identifications of orthogonal cutting coefficients. Wang [6] conducted an examination of the fundamental mechanics of cutting force coefficients. Zhao [7] optimized the cutting conditions for constant cutting forces during milling. Gradzka and others examined the coefficients of cutting forces to determine vibrations [8]. They found that this method can be used for the experimental determination of cutting forces depending on vibration. Kuruc [9] compared high-feed machining with conventional milling in terms of dimension, accuracy and productivity. It was confirmed that high-feed milling (HFM) can achieve considerable machining time reduction and is therefore suitable for roughing. Wang [6] studied the dependence of cutting forces on changing cutting conditions and determined their coefficients in the X and Y axes. The cutting force coefficients derived from the average cutting force model depend on the tool–workpiece material couple. For the same axial cutting depth, the milling force values in the X and Y directions were roughly the same; i.e., varying the spindle speed does not change the magnitude of the cutting force.

Another author that dealt with the cutting forces was Tukora [10], who made a cutting-force-predicting method based on the mechanistic cutting force. The proposed way of determining the cutting force coefficients makes it possible to perform coefficient determination and force prediction simultaneously. Zhu [11] followed up on previous work and analyzed the characteristics of previously created models and proposed an experimental method for selecting a suitable cutting force model for a real cutting process. Pa [1,12] investigated the effect of cutting forces on residual stress. He found that the accuracy of the analysis of the cutting process dynamics was significantly affected by the cutting force. Jiang [13] made a dynamic milling force model for a milling cutter under vibration. His model showed that due to the influence of milling vibration and cutter tooth error, the milling cutter had a constantly changing displacement increment in three directions of the workpiece. Tsai [14] investigated cutting forces during milling of an aluminum alloy. Bari [15] performed a comparative analysis of cutting forces and stability of serrated mills with standard and non-standard profiles. Wu [16] worked on cutting force prediction for the circular end milling process. Li [17,18] dealt with cutting power and power efficiency during the straight-tooth cylindrical milling process of particle boards and modeling and predicting the machined surface roughness and milling power in Scot’s pine helical milling process. Sousa [19] provided an overview of a data acquisition system for cutting force measuring and optimization in milling.

This research paper is focused on the influence of cutting conditions on the decomposition of the cutting forces in the terms of their size and ratios in the three main axes, X, Y and Z. Understanding how this decomposition occurs can help with the optimization of cutting conditions and thus a reduction in production time.

## 2. Materials and Methods

### 2.1. Tested Material

The chosen material was 1.4541, which is a titanium-stabilized austenitic stainless steel with good corrosion resistance (Table 1, Table 2 and Table 3). It has excellent resistance to intergranular corrosion after exposure to temperatures in the chromium carbide precipitation range of 427–816 °C. The alloy resists oxidation up to 1500 °F (816 °C) and has higher creep and stress rupture properties than alloys 304 and 304 L. This material is suitable for turbines, scoop wheels and parts exposed to continual centrifugal stress. It also possesses good low-temperature toughness [20].

### 2.2. Machine Tool and Tools

**Figure 1:**
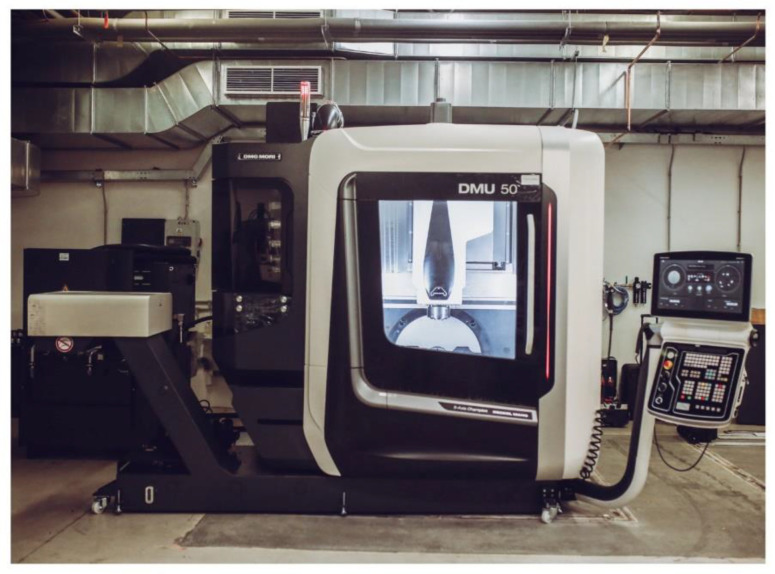
Five-axis vertical milling center DMU 50.

The universal CNC milling machine from DMG Mori allows machining in five independent axes (Figure 1). The machine spindle allows movement in the X, Y and Z axes. The machine table enables rotation in the B and C axes, where the range in the B axis is −35/+110°. The maximum spindle speed of the machine is 15,000 rpm, and the rapid transverse speed is 30 m/min.

Cermet inserts (LPHT060310TR-M06 MS2050) from SECO have a width of 6.4 mm and a thickness of 3.18 mm. There are two cutting edges with a face angle of 11°. The plates are treated with a special MS2050-class PVD coating [21].

The milling head from SECO allows the clamping of three inserts. The machining diameter of the milling head is 20 mm, and the maximum depth of cut is 0.8 mm. The functional length of the head is 28 mm [22].

### 2.3. Milling Force Measuring

A dynamometer is a measuring device that is used to measure cutting forces (dynamic loads) during machining. The Kistler Type 9129AA Dynamometer has a range from −10,000 N to 10,000 N and the resolution is 0.1 N. The functional dimensions of the Dynamometer are 90 × 105 × 32 mm (length × width × height), and the total length is 150 mm.

**Figure 2:**
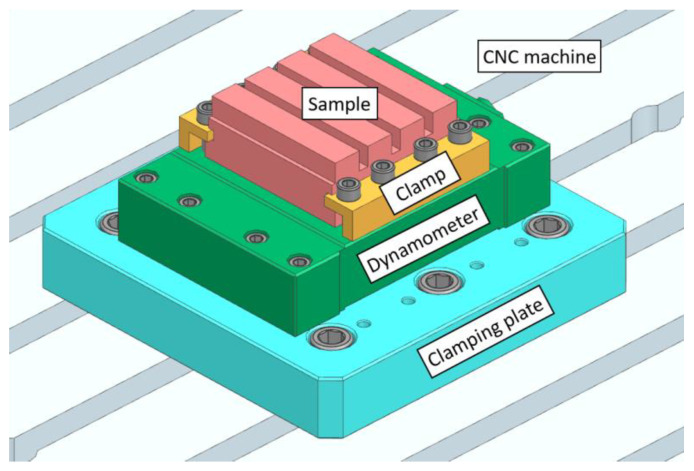
Clamping of the specimen to the dynamometer and CNC machine.

Figure 2 shows the attachment of the sample to the dynamometer by means of clamps and Allen screws. The dynamometer itself was attached to the clamping plate, which was attached to the table of the CNC milling machine with the help of Allen screws.

**Figure 3:**
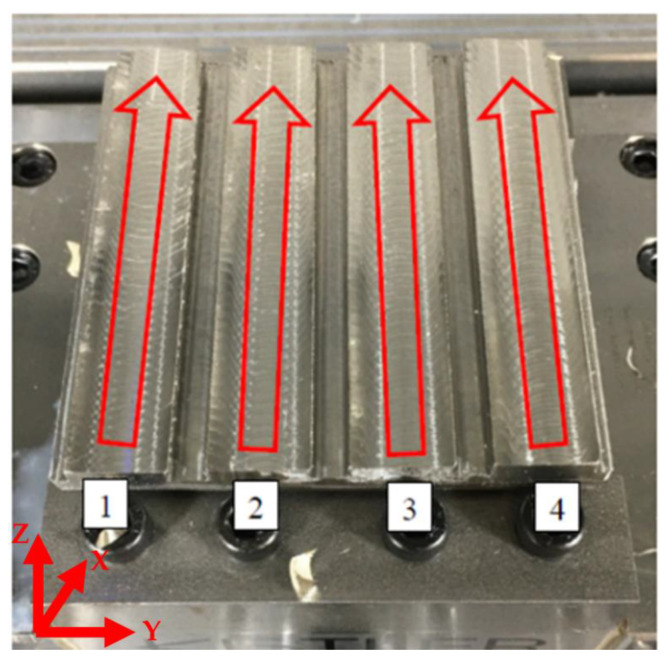
Manner of machining and orientation of cutting forces.

Plane milling was chosen for the experiment, in which four surfaces are machined in one series at a time. The width of the machined surface was 14 mm. Length of cut was 74 mm in the main cutting direction. Due to elimination of interfering forces that could influence the value of the scanned data, grooves with a 5 mm thickness were milled into the sides. This enabled easy transition to and out of the cutting process. The cutting conditions changed during milling. The cutting speed (vc), depth of cut (ap) and feed per tooth (fz) varied. The workpiece was clamped on a dynamometer during milling to measure the forces Fx, Fy and Fz acting on the workpiece. After milling the given surfaces, the workpiece was unclipped from the dynamometer and the surface quality parameters, i.e., Ra, Rz and Rt, were measured. Figure 3 shows the orientation of the cutting forces on a Kistler 9129AA dynamometer. 

Figure 3 shows planar milling sections made during the experiment. The red arrows indicate the direction of the tool movements during machining at the selected parameters. Each number indicates different cutting conditions.

The experiment was conducted precisely in the following manner: the tool was placed 10 mm above the surface intended for machining, recording of cutting forces was turned on and preset feed of the cutting tool was enabled. The experiment ended when the tool reached 10 mm behind machined surface. 

Table 4 shows the cutting conditions used in the experiment. The mean value of these selected conditions corresponds to the tool manufacturer’s recommendation for the machined material used. Values were further increased/decreased to obtain a range of parameters, thereby creating dependence curves or trends of machining. The width parameter, ae, was kept constant because of the diameter of the tool used. Each experiment was carried out 10 times for each parameter set. Afterwards, the mean arithmetic value of force was calculated from the obtained data. 

## 3. Results

Table 5 shows the cutting forces recorded in DynoWare as the course of force over time. This course created ten non-overlapping intervals, from which the maximum values Fx, Fy and Fz were selected and entered into the tables. From these values, the average values were calculated.

When analyzing the values listed in Table 5, the influence of the displacement per tooth on the absolute values of the individual force components was noticeable. For all displacement parameters, the force Fy was the most significant value, and the force Fx was the least significant. However, to analyze the load of the entire machining system, it was also necessary to determine its total loading force, Fc. The calculation size of this force is given by the vector sum of the individual forces according to Formula (1). From the measured data and the formula used, it can be concluded that the component Fy will have the largest share of the resulting force, followed by Fz, while the component Fx will have the smallest share of the resulting force Fc.

(1) $$F_{c}=\sqrt{F_{x}^{2}+F_{y}^{2}+F_{z}^{2}}$$

The calculation of the resulting force mentioned above is important from the point of view of the total load on the tool edge and the determination of the final forces acting on the tools or on the workpiece. However, individual comparison of absolute values does not allow excessive generalization when the individual values of the components of the resulting force are related to the individual components of the total force. Therefore, new parameters were introduced with the designations Fxp, Fyp and Fzp, which represent the relative percentage of the effect of the individual components on the resulting Fc strength. A simple example of calculating the Fxp component can be seen in Formula (2).

(2) $$Fxp=\frac{F_{x}}{F_{c}}*100\left(\%\right)$$

Table 6 shows the individual percentages of the force components acting on the resulting force. Although this conversion eliminates the possibility of determining the absolute load for individual measurement directions, it allows improved interpretation of the change in direction of measured forces, their share in total applied force Fc and their mutual comparison.

**Figure 4:**
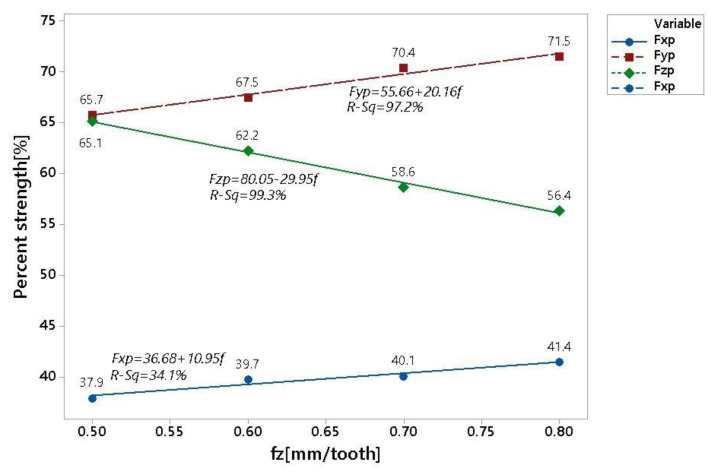
Graphical representation of cutting force ratios.

For a better interpretation of the calculated data in Table 6, the graph presented in Figure 4 was drawn up. From this graph, it is clear that for the engagement depth ap = 0.5 mm in the measured displacement range of 0.5–0.8 mm/tooth, the Fxp component had the smallest increase. A stronger trend can be seen in the Fyp curve, where the share increased by approximately 6%. On the contrary, the decline in the Fzp component was approximately 9%.

The displayed results were further statistically examined using linear regression, where the individual points were interpolated with a straight line, the parameters of which can be found in Table 7. The displacement value along the vertical axis is expressed by the parameter Abs, and the angle of inclination of the interpolated line is expressed using the parameter f.

Other data from measurements for ap 0.6 and 0.7 mm were subjected to the same method of data evaluation as above. Results can be seen in Table 8 and Table 9 listed below.

When comparing the results for cutting forces at a constant cutting speed, it is evident that the cutting forces increased with increasing depth of cut. The percentage of forces from the vector sum varied very similarly for all the aforementioned results. 

**Figure 5:**
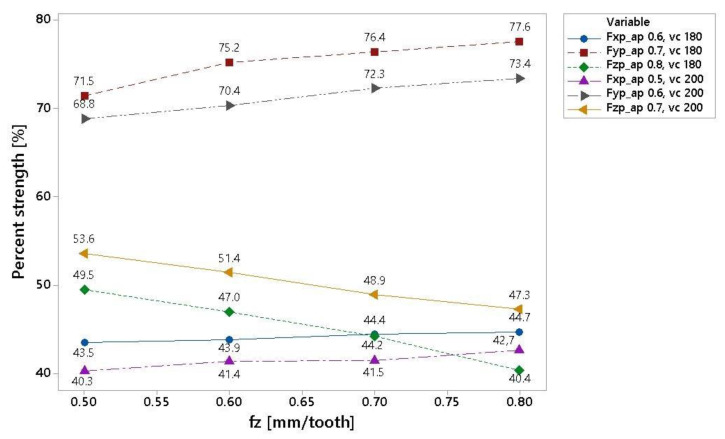
Graphical representation of Fz with varying cutting conditions.

Figure 5 shows a graphic display of the result for various cutting conditions. As in Figure 4, the force Fy had the largest percentage, and its value increased with increasing tooth feed. The Fx forces had the lowest initial values. This graph shows that the force trends were the same for all cutting conditions. 

**Figure 6:**
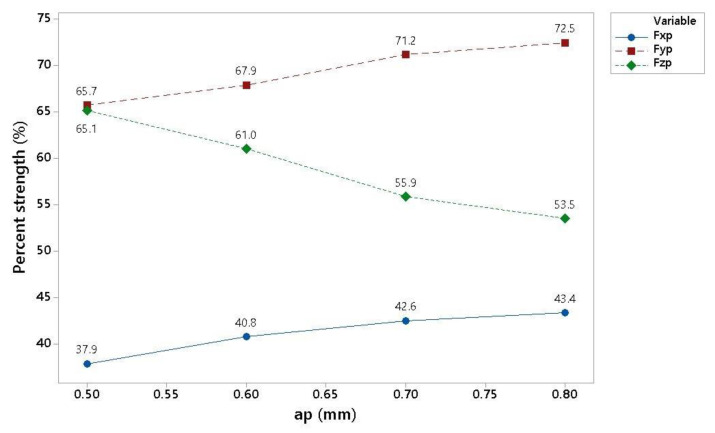
Graphical representation of fz = 0.6 mm/tooth and vc = 180 mm/min.

When comparing different machining cutting conditions at a cutting depth, ap, of 0.5 to 0.8 mm at a cutting speed of 180 m/min and 200 m/min (Figure 5), very similar trends in the relative representation of individual forces (Fx, Fy, Fz) can be seen in components of the resulting force. The influence of the depth of cut can be observed in Figure 6. As can be seen, increasing chip size led to a decrease in the axial stress of the tool and an increase in the radial load of the tool. There was a more pronounced effect on the size of the Fy component, which acted tangentially to the section. As can be seen from the Fx forces data, approximately half of the influence acted normally against the cut. Another trend that can be seen in the figure is a change in the cutting speed. An increase in the cutting speed from 180 m/min to 200 m/min caused a decrease in the share of the Fx and Fy components in the total force and an increase in the Fz component. This change was also observed in a similar test that uses the melting of the material at the point of the cut, thereby reducing the resistance to the penetration of the cutting wedge.

All these effects significantly influence the creation of chips, especially in the primary chipping area, which is in contact with the tool. The most important aspects of chip theory are the creation of heat on the front of the tool and the shaping of the chip. The total value of this heating is the result of numerous effects that can be mutually replaced while keeping the gains of heat energy. A question that needs to be answered is the possibility of replacing one component with another.

The results of cutting forces depending on the feed per tooth at a variable depth of cut are presented above. A strong statistical dependence was found on the obtained data. It is, therefore, possible to derive from these values with a high degree of probability what forces would be measured when changing the cutting parameters.

This time, the results will be presented as dependence on the depth of cut (Table 10).

During machining, it is possible to increase productivity by increasing feed per tooth. It is also possible to increase the cross-section of the chip by the depth of engagement ap. The absolute measurement of the value listed in Table 11 for a feed of 0.5 mm/tooth and a cutting speed of 160 m/min with a depth of cut of 0.5 mm to 0.8 mm can be seen in Figure 6. Here, the same trends can be observed for the individual components of the resulting force, but with a more pronounced effect for the component in the direction of the z axis. This effect can be caused by the influence of the geometry of the tool, which has a 1 mm radius of the rounding. Thus, the cutting tool engagement in the material changes as the depth of engagement gradually increases.

A change in components in total force for individual depths of cut can significantly influence the choice of strategy in part machining, especially for parts that are easily deformed and damaged. Based on this information, the programmer can choose a suitable direction of machining in order to stress the part as little as possible (Table 12).

**Figure 7:**
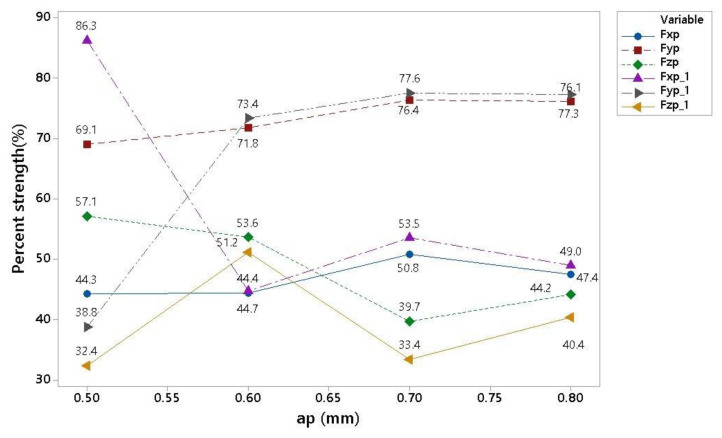
Graphical comparison of the results for two cutting conditions.

The results for a 180 m/min cutting speed, a feed per tooth of 0.5 mm and a depth of cut of 0.5 to 0.8 mm are shown in Table 13 and Figure 7. The data are ambiguous between 0.5 mm and 0.6 mm and show no trend. Even with repeated measurements, the data showed random phenomena. In a more detailed analysis, the inhomogeneous data were attributed to the boundary process conditions, especially the cutting speed of 180 m/min in connection with the depth of cut of 0.5 mm and the feed per tooth of 0.5 mm. These conditions no longer allowed homogeneous chip formation conditions for the given material or the formation of a melted area of the machined material on the cutting tool’s edge.

**Figure 8:**
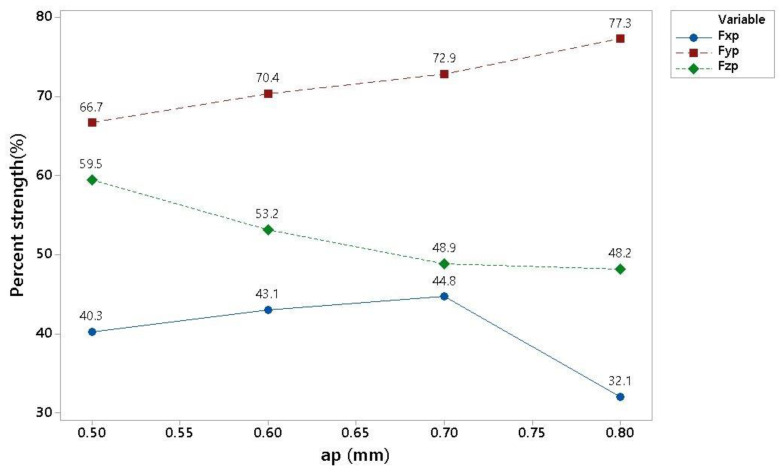
Graphical display of the results for various cutting conditions.

Figure 8 compares two sets of results for different cutting conditions, specifically vc = 180 mm/min f = 0.7/0.8 mm and ap variates from 0.5 to 0.8 mm.

The theory that a small load on the tool edge will lead to insufficient conversion of mechanical energy into thermal energy occurring at the cutting point was subsequently confirmed by an increase in the cutting speed from 180 m/min to 200 m/min while maintaining other machining parameters. The resulting breakdown of the forces can be seen in Figure 8. Here, it can be seen that the process was stable even for an engagement depth range of 0.5 to 0.6 mm. The decrease in the trend of the relative strength Fx for ap 0.8 was not explained despite repeated measurements; therefore, it is worth further investigation. Future research could, for example, focus on the case in which the mechanical properties of the machined material are not exceeded.

## 4. Conclusions

In this paper, an experiment was performed to find out how the cutting forces decompose during milling, and if this decomposition can be predicted. In this experiment, a titanium-stabilized austenitic stainless steel (1.4541) was used. The machining was conducted on DMU 50. 

Three cutting speeds (160, 180 and 200 mm/min) were used. At these speeds, machining took place at four different depths of cut at four different feeds per tooth. 

The results of cutting forces were averaged, the vector product was calculated, and the percentage shares of forces in three axes were calculated from it, namely X, Y and Z. As expected, the load increased with increasing depth of cut and feed per tooth. However, there was an interest in examining the percentage shares of cutting forces. 

The results of these shares are very similar; for example, Fxp for vc = 160 m/min, ap = 0.5 mm and fz = 0.6 mm/tooth was 39.7 and Fxp for vc = 200 m/min, ap = 0.6 mm and fz = 0.6 mm/tooth was 38.2. 

It was confirmed that the percentage of force Z decreases with increasing load for vc = 180 m/min, ap = 0.8 mm and fz = 0.6 to 0.8 mm/tooth. The share decreased from 49.5 to 40.4. 

A statistical dependence of the proportion of forces on the tooth displacement was proven. This proves that it is possible to predict the load of a milling tool in its basic axes. 

## Figures and Tables

**Table 1:** Chemical composition of 1.4541 [20].

Element	Content (%)
C	0.04
Si	0.40
Mn	1.20
Cr	17.50
Ni	9.50
Mo	2.10
Ti	0.35

**Table 2:** Physical properties of 1.4541 (20 °C) [20].

Property	Unit	Value
Density	(g/cm^3^)	7.9
Specific heat capacity	(J/kg K)	500
Thermal conductivity	(W/mK)	15
Electrical resistivity	(Ω mm^2^/m)	0.73

**Table 3:** Mechanical properties of 1.4541 (20 °C) [20].

Property	Unit	Value
Hardness HB 30	(HB)	205
0.2% Yield strength Rp	(N/mm^2^)	195
Tensile strength Rm	(N/mm)	593
Elongation A_5_	(%)	40/30
Modulus of elasticity	(kN/mm^2^)	200

**Table 4:** Cutting conditions of the experiment.

Sample Number	Ae (mm)	vc (m/min)	ap (mm)	fz (mm/tooth)
Sample 1	14	160	0.5	0.5
Sample 2	14	160	0.5	0.6
Sample 3	14	160	0.5	0.7
Sample 4	14	160	0.5	0.8
Sample 5	14	160	0.6	0.5
Sample 6	14	160	0.6	0.6
Sample 7	14	160	0.6	0.7
Sample 8	14	160	0.6	0.8
Sample 9	14	160	0.7	0.5
Sample 10	14	160	0.7	0.6
Sample 11	14	160	0.7	0.7
Sample 12	14	160	0.7	0.8
Sample 13	14	160	0.8	0.5
Sample 14	14	160	0.8	0.6
Sample 15	14	160	0.8	0.7
Sample 16	14	160	0.8	0.8

**Table 5:** Average value of cutting force in terms of dependence on feed.

ap 0.5 (mm)			
vc 160 (m/min)			
fz (mm/tooth)	Fx (N)	Fy (N)	Fz (N)
0.5	346.04	600.09	594.69
0.6	406.96	690.68	636.94
0.7	454.80	798.73	665.00
0.8	513.71	885.99	698.62

**Table 6:** Percentages of forces.

ap 0.5 (mm)			
vc 160 (m/min)			
fz (mm/tooth)	Fxp (%)	Fyp (%)	Fzp (%)
0.5	37.9	65.7	65.1
0.6	39.7	67.5	62.2
0.7	40.1	70.4	58.6
0.8	41.4	71.5	56.4

**Table 7:** Results of linear regression.

	Fxp (%)	Fyp (%)	Fzp (%)
Abs	32.7	55.6	79.9
f	10.9	20.3	−29.7

**Table 8:** Results for ap = 0.6 (mm) and vc = 160 (m/min).

ap 0.6 (mm)						
vc 160 (m/min)						
Measured Data				Results		
f_z_ (mm/z)	Fx (N)	Fy (N)	Fz (N)	Fxp (%)	Fyp (%)	Fzp (%)
0.5	408.14	679.16	610.45	40.8	67.9	61.0
0.6	466.07	794.23	645.04	41.5	70.6	57.4
0.7	526.88	903.53	679.3	42.2	72.4	54.5
0.8	578.75	972.57	684.26	43.8	73.5	51.7

**Table 9:** Results for ap = 0.7(mm) and vc = 160 (m/min).

ap 0.7 (mm)						
vc 160 (m/min)						
Measured Data				Results		
f_z_ (mm/tooth)	Fx (N)	Fy (N)	Fz (N)	Fxp (%)	Fyp (%)	Fzp (%)
0.5	472.38	790.18	620.45	42.6	71.2	55.9
0.6	538.93	901.59	672.41	43.2	72.3	53.9
0.7	608.03	1035	715.67	43.5	74.1	51.2
0.8	672.16	1132	739.99	44.5	75.0	49.0

**Table 10:** Results for ap = 0.7 mm and vc = 160 m/min.

ap 0.7 (mm)						
vc 160 (m/min)						
Measured Data				Results		
f_z_ (mm/tooth)	Fx (N)	Fy (N)	Fz (N)	Fxp (%)	Fyp (%)	Fzp (%)
0.5	397.86	971.42	659.20	32.1	78.4	53.2
0.6	511.3	1034.97	677.14	38.2	77.3	50.6
0.7	615.36	1113.3	700.1	42.4	76.7	48.2
0.8	772.63	1236.8	753.59	47.1	75.3	45.9

**Table 11:** Results for fz = 0.5 mm/tooth and vc = 160 m/min.

fz 0.5 (mm/tooth)						
vc 160 (m/min)						
Measured Data				Results		
ap (mm)	Fx (N)	Fy (N)	Fz (N)	Fxp (%)	Fyp (%)	Fzp (%)
0.5	346.04	600.09	594.69	37.9	65.7	65.1
0.6	406.96	690.68	636.94	40.8	67.9	61.0
0.7	454.8	798.73	665	42.6	71.2	55.9
0.8	513.71	885.99	698.62	43.4	72.5	53.5

**Table 12:** Results for fz = 0.8 mm/tooth and vc = 160 m/min.

fz 0.8 (mm/tooth)						
vc 160 (m/min)						
Measured Data				Results		
ap (mm)	Fx (N)	Fy (N)	Fz (N)	Fxp (%)	Fyp (%)	Fzp (%)
0.5	520.12	869.19	641.92	41.4	71.5	56.4
0.6	620.43	1000.72	692.98	43.8	73.5	51.7
0.7	700.04	1148.7	734.73	44.5	75.0	49.0
0.8	733.06	1197.2	724.96	46.9	75.5	45.7

**Table 13:** Results for fz = 0.5 mm/tooth and vc = 180 m/min.

fz 0.5 (mm/tooth)						
vc 180(m/min)						
Measured Data				Results		
ap (mm)	Fx (N)	Fy (N)	Fz (N)	Fxp (%)	Fyp (%)	Fzp (%)
0.5	291.14	202.23	168.92	74.1	51.5	43.0
0.6	447.16	695.54	611.59	43.5	69.5	57.2
0.7	497.34	776.35	641.82	42.9	71.5	55.2
0.8	372.01	167.46	139.59	45.1	74.3	49.5

## Data Availability

The data presented in this study are available on request from the corresponding author.
